# Orthogonal Decomposition of the Genetic Variance for Epistatic Traits Under Linkage Disequilibrium—Applications to the Analysis of Bateson-Dobzhansky-Müller Incompatibilities and Sign Epistasis

**DOI:** 10.3389/fgene.2019.00054

**Published:** 2019-03-05

**Authors:** José M. Álvarez-Castro, Rosa M. Crujeiras

**Affiliations:** Department of Statistics, Mathematical Analysis and Optimization, Universidade de Santiago de Compostela, Santiago de Compostela, Spain

**Keywords:** genetic variance decomposition, linkage disequilibrium, epistasis, Bateson-Dobzhansky-Müller incompatibilities, sign epistasis

## Abstract

The one-century-old theory of orthogonal genetic variance decomposition originated the field of quantitative genetics and has kept on being improved ever since. Recently, serious concerns about the possibility of attaining a satisfactory implementation of genetic variance decomposition with linkage disequilibrium (LD) and epistasis have been raised. In this paper we dissipate such doubts by completing the classical theory of variance decomposition into additive, dominance and epistasis components with LD. We apply that theory to the analysis of the genotype-to-phenotype maps of two cases of particular evolutionary interest—Bateson-Dobzhansky-Müller incompatibilities and sign epistasis. For the first case we show how negative LD and reduction of heterozygotes may contribute to maintain genetic variability after secondary contact. For the second case we show that LD transforms the set of frequencies leading to an evolutionary plateau into a ridge. Our theoretical developments reassuringly reflect the complexity LD conveys to genetic systems throughout novel properties—as compared with systems under linkage equilibrium. We argue that such particularities might have actually contributed to cause confusion about the feasibility of developing this methodology. In any case, the theory we provide in this paper enables new perspectives in both evolutionary and quantitative genetics studies.

## Introduction

Genetic variance decomposition has gained increased scientific attention one century after it was first developed by Fisher (1918). At that time, that theory was necessary for denying that Mendelian inheritance could be in contradiction with Galton's (1886) regression toward mediocrity in traits with continuous variation. Beyond that, variance decomposition endorsed regression toward mediocrity with a mechanistic explanation and provided a satisfactory genetic basis to Darwinian gradual evolution (Provine, 1971).

With time, models of genetic effects enabling variance decomposition (and thus disentangling resemblance between relatives) in the face of increasingly complex genetic systems and/or population facts were developed. A historical, key step forward was given by Kempthorne (1954) and Cockerham (1954) in the year following the publication of the double helix structure of DNA (Franklin and Gosling, 1953; Watson and Crick, 1953), by providing extensions to multiple alleles and epistasis, on the one hand, and to Hardy-Weinberg disequilibrium (HWD) in a two-locus two-allele epistatic system, on the other hand, respectively. Still, the practical use of those implementations was subject to apparent limitations at the time when the exponential growth of molecular biology had just been kicked off.

More recently, the development of molecular genetics has made it possible to obtain datasets large enough to undertake realistic strategies of genetic mapping and genomic prediction. Consequently, along the latest quarter century models of genetic effects and variance decomposition have been thoroughly revisited (e.g., Cheverud and Routman, 1995; Hansen and Wagner, 2001; Yang, 2004; Zeng et al., 2005; Mao et al., 2006; Alvarez-Castro and Carlborg, 2007; Álvarez-Castro and Yang, 2011, 2015; Ma et al., 2012; Álvarez-Castro, 2014; Xiao et al., 2014). However, among all possible implementations, linkage disequilibrium (LD) has not yet been satisfactorily addressed—more to the point, it has even been claimed to be unfeasible (see Vitezica et al., 2017 and references therein).

In this paper, we provide theoretical developments enabling the decomposition of the genotypic values and the genetic variance with arbitrary numbers of loci and alleles, with any kind of dominance and epistatic interactions and with arbitrary population frequencies—i.e., under arbitrary departures both from Hardy-Weinberg equilibrium (HWE) and from linkage equilibrium (LE). We also provide applications of our methodology to two cases of special evolutionary interest—Bateson-Dobzhansky-Müller (BDM) incompatibilities and sign epistasis—and review and discuss arguments on which doubts about the feasibility of a genetic decomposition with LD and epistasis were based.

## Genetic Variance Decomposition

### Conceptual Background

Following Fisher's (1918, 1930) scheme, the decomposition of the genetic variance can be defined as a property of a particular genetic system at a particular population, which mathematically translates into a function whose variables are the genotypic values (the expected phenotype of each genotype) and the population genotypic frequencies. That function provides the proportion of genetic variance attributable to each genetic component (additive, dominance, epistasis, and imprinting) into which the genotypic values can be split—i.e., reparameterized using a mathematical model. The additive component is the one associated to the most important and intuitive biological interpretation—it enables the analysis of resemblance between parents and offspring involved in the concept of narrow-sense heritability. Nevertheless, the remaining components of variance are also biologically meaningful since they enable the analysis of further instances of resemblance between relatives and various evolutionary interpretations (see e.g., Kempthorne, 1957; Álvarez-Castro, 2014; Alvarez-Castro and Le Rouzic, 2015). Imprinting is though out of the scope of this paper.

### Mathematical Model

The classical (statistical) genetic model for a locus *A* with two alleles *A*_1_ and *A*_2_ decomposes the genotypic values—i.e., the expected phenotype of each genotype, *G* = (*G*_11_, *G*_12_, *G*_22_)—into additive and interaction (dominance) components using the linear regression framework *G* = **1**μ+**N**α+δ (Kempthorne, 1957), which can be expanded as

(1) $$\left(\begin{array}{c} G_{11}\\ G_{12}\\ G_{22} \end{array}\right)=\left(\begin{array}{c} 1\\ 1\\ 1 \end{array}\right)\mu +\left(\begin{array}{cc} 2 & 0\\ 1 & 1\\ 0 & 2 \end{array}\right)\left(\begin{array}{c} \alpha_{1}\\ \alpha_{2} \end{array}\right)+\left(\begin{array}{c} \delta_{11}\\ \delta_{12}\\ \delta_{22} \end{array}\right),$$

where μ is the population mean phenotype, **N** indicates the number of alleles of each type in each genotype, α_*i*_, *i* = 1,2 (the explanatory variables), are the average (additive) effects of the alleles and δ_*ij*_, *j* = 1,2, *i* ≤ *j*, (the error terms) are the dominance deviations.

The regression model in Equation (1) is solved by first clearing away the term with the mean phenotype using the mean-corrected genotypic values as

(2) $$\begin{array}{r} \bar{G}=G-1\mu \end{array}$$

and then solving the resulting regression $\bar{G}$ = **N**α +δ. The weighted least-squares (WLS) solution and error terms for this regression may be obtained from its normal equations, **N**'**PN**α = **N**'**P**$\bar{G}$, where **N** is called the design matrix of the regression, **N**' stands for its transpose and **P** (called the weights matrix) contains the population genotypic frequencies (*f* (*A*_*i*_*A*_*j*_) = *p*_*ij*_, *j* = 1,2, *i* ≤ *j*) in its diagonal, i.e., **P** = diag(*p*_*ij*_) (the theory of matrix algebra applied to linear regression used in this paper comes from Harville, 1997; Draper and Smith, 1998). This way we obtain

(3) $$\begin{array}{r} \alpha =\tilde{\text{H}}\bar{G},\text{ with }\tilde{\text{H}}={\left({\text{N}}^{\prime }\text{P}\text{N}\right)}^{-1}{\text{N}}^{\prime }\text{P}. \end{array}$$

Matrix **H** = **N**$\tilde{\text{H}}$ is often called the hat matrix of the regression and **M** = **I**–**H** (**I** being the identity matrix with the proper dimension, 3 × 3) is the annihilation matrix. Using the latest matrix the error terms may be equated as

(4) $$\begin{array}{r} \delta =\text{M}\bar{G}. \end{array}$$

It is worth noting that the regression model in Equation (1), as solved above, is not equivalent to *G* = (1|**N**)(μ|α)'+δ expanding to

(5) $$\left(\begin{array}{c} G_{11}\\ G_{12}\\ G_{22} \end{array}\right)=\left(\begin{array}{c} 1\\ 1\\ 1 \end{array}|\begin{array}{cc} 2 & 0\\ 1 & 1\\ 0 & 2 \end{array}\right)\left(\begin{array}{c} \mu \\ \begin{array}{c} \bar{\alpha_{1}}\\ \alpha_{2} \end{array} \end{array}\right)+\left(\begin{array}{c} \delta_{11}\\ \delta_{12}\\ \delta_{22} \end{array}\right).$$

In fact, the WLS solution of the regression in Equation (5) can easily be found to be different from the results obtained within Equations (2–4). More to the point, Equation (2) can actually be obtained from the WLS solution of regression *G* = **1**μ+η. In this expression, vector **1** = (1, 1, 1)′ is the design matrix, **11**′**P** is the hat matrix, μ = **1**′**P***G* is the explanatory variable, **I**–**11**′**P** is the annihilation matrix and η = (**I**–**11**′**P**)*G* are the error terms. Hence, the mean-corrected vector of genotypic values $\bar{G}=$ *G*–**1**μ in Equation (2) can actually be interpreted as the error terms of regression *G* = **1**μ+η, since the WLS solution of that regression provides the error terms as η = (**I**–**11**′**P**)*G* = *G*–**1**μ.

Overall, for obtaining the biologically meaningful parameters aimed in the regression model in Equation (1), the solution is not achieved in a single step—i.e., is not achieved as by means of Equation (5). It is instead achieved in two regression steps, the first of which is *G* = **1**μ+η, evidently leading to η = $\bar{G}$, and the second of which is η = $\bar{G}$ = **N**α+δ. Below it will be made clear that the methodology provided in this paper for decomposing the genotypic values and the genetic variance in the face of LD and epistasis fits squarely with this sequential procedure.

### Orthogonal Variance Decomposition

Using the solution to Equation (1) obtained above within Equations (2–4), the genotypic values may be decomposed as:

(6) $$\begin{array}{r} G_{ij}=\mu +\alpha_{ij}+\delta_{ij},\;j=1,2,i\;\leq \;j, \end{array}$$

where, for each genotypic value, *G*_*ij*_, its additive component is α_*ij*_ = α_*i*_+α_*j*_—cf Equation (1). These α_*ij*_ are actually the breeding values, at least under HWE. In any case, the decomposition of the genotypic values in Equation (6) directly provides the corresponding decomposition of the genetic variance, which can be given by the variance of the values obtained for each set of components (e.g., Bürger, 2000) as

(7) $$\begin{array}{r} {\text{V}}_{\text{A}}=\text{V}\left(\alpha_{ij}\right),\;{\text{V}}_{\text{D}}=\text{V}\left(\delta_{ij}\right). \end{array}$$

Since the above decomposition of the genotypic values (Equation 6) is orthogonal by construction, the variance components in Equation (7) provide an accurate decomposition of the genetic variance, V_G_ = V(*G*_*ij*_) = V_A_+V_D_, and hold their biological interpretations. Orthogonality is also an extremely useful statistical property for the development of appropriate model selection strategies in genetic mapping studies, as resumed in the discussion.

The mathematical model in Equation (1) has recently been extended to an arbitrary number of alleles with arbitrary HWD (Álvarez-Castro and Yang, 2011). It can also be extended to accommodate an arbitrary number of (multiallelic) loci, and shall then involve an epistasis component. Although the developments currently available may account for arbitrary epistasis, they attain orthogonality (and thus are accurate in what regards their biological meaning) only when no departures from LE frequencies occur (see Álvarez-Castro and Yang, 2011; Vitezica et al., 2017 and references therein). In what follows, we provide new multilocus extensions of the mathematical model in Equation (1) that do not assume LE, thus holding orthogonality in the face of arbitrary population frequencies—as well as arbitrary interactions within and between/among loci.

## Theoretical Results

### Mean and Additive Component

We now consider an additional biallelic locus, *B*, with alleles *B*_1_ and *B*_2_. We start by detaching the mean and the additive component of the model in a way analogous to regression (Equation 1) above. Therefore, we consider regression *G* = **1**μ+**N**_α_α+η_α_, expanding to

(8) $$\left(\begin{array}{c} G_{1111}\\ G_{1211}\\ G_{2211}\\ G_{1112}\\ G_{1212}\\ G_{2212}\\ G_{1122}\\ G_{1222}\\ G_{2222} \end{array}\right)=\left(\begin{array}{c} 1\\ 1\\ 1\\ 1\\ 1\\ 1\\ 1\\ 1\\ 1 \end{array}\right)\mu +\left(\begin{array}{llll} 2 & 0 & 2 & 0\\ 1 & 1 & 2 & 0\\ 0 & 2 & 2 & 0\\ 2 & 0 & 1 & 1\\ 1 & 1 & 1 & 1\\ 0 & 2 & 1 & 1\\ 2 & 0 & 0 & 2\\ 1 & 1 & 0 & 2\\ 0 & 2 & 0 & 2 \end{array}\right)\left(\begin{array}{l} \alpha_{1}^{A}\\ \alpha_{2}^{A}\\ \alpha_{1}^{B}\\ \alpha_{2}^{B} \end{array}\right)+\left(\begin{array}{c} \eta_{1111}^{\alpha }\\ \eta_{1211}^{\alpha }\\ \eta_{2211}^{\alpha }\\ \eta_{1112}^{\alpha }\\ \eta_{1212}^{\alpha }\\ \eta_{2212}^{\alpha }\\ \eta_{1122}^{\alpha }\\ \eta_{1222}^{\alpha }\\ \eta_{2222}^{\alpha } \end{array}\right)$$

The rows of **N**_α_ are just all possible combinations of two rows of the one-locus matrix **N** in Equation (1). In any case, in a first step we obtain Ḡ = *G*–**1**μ, as in Equation (2) for the one-locus case. Then, the second step consists in obtaining the additive component as the WLS solution of regression Ḡ = **N**_α_α+η_α_. Equations (3, 4) and related text would typically provide the WLS solution for this case as well, did our new matrix **N**_α_ in the regression model in Equation (8) not lead to a singular matrix **N**_α_′**PN**_α_. However, it actually does. Conveniently, this issue may be overcome by performing the regression outside the kernel, as follows.

First, the eigenvalues and eigenvectors of singular matrix **N**_α_′**PN**_α_ are computed—in practice, this is done simply using e.g., the appropriate built-in commands of R Core Team (2017). Next, a diagonal matrix, **D**_α_ is built with the non-nil eigenvalues so obtained, while their corresponding eigenvectors become the columns of matrix **U**_α_. Then, the solution may be obtained as in Equation (3), using in this case the matrices obtained just above, as

(9) $$\alpha ={\tilde{\text{H}}}_{\alpha }\bar{G},\text{ with }{\tilde{\text{H}}}_{\alpha }={\left({\text{U}}_{\alpha }{\left({\text{D}}_{\alpha }\right)}^{-1}{\text{U}}_{\alpha }{\;}^{\prime }\right)}^{-1}{\text{N}}_{\alpha }{\;}^{\prime }\text{P}.$$

The error term is then obtained, as in Equation (4), as

(10) $$\begin{array}{r} \eta_{\alpha }={\text{M}}_{\alpha }Ḡ, \end{array}$$

using the hat matrix **H**_α_ = **N**_α_${\tilde{\text{H}}}_{\alpha }$ and its corresponding annihilation matrix **M**_α_ = **I**–**H**_α_, being understood that the identity matrix **I** is used in its appropriate dimension, which for this case is 9 × 9.

The extension of the regression model in Equation (8) to *l* multiple biallelic loci is straightforward, by just enlarging the rows of regression matrix **N**_α_ to accommodate all combinations of *l* repetitions of the rows of regression matrix **N** in Equation (1). Complexity may also be increased straightforwardly in what regards the numbers of alleles, by just building **N**_α_ from single-locus design matrices that, as opposed to **N** in Equation (1), are appropriate for the number of alleles, *n*_*j*_, of each locus, *j*. As mentioned above, such matrices have already been provided (Álvarez-Castro and Yang, 2011).

For the case of two biallelic loci, it is easy to derive from the design matrix **N**_α_ in Equation (8) that the additive components of the genotypic values are α_*ijkl*_ = $\alpha_{i}^{A}+\alpha_{j}^{A}+\alpha_{k}^{B}+\alpha_{l}^{B}$, *j* = 1,2, *i* ≤ *j, l* = 1,2, *k* ≤ *l*. In the general case (with *n*_*j*_ alleles in each locus *j, j* = 1,…,*l*) we would have α_*G*_ = $\overset{l}{\underset{j=1}{\sum }}\overset{n_{j}}{\underset{i=1}{\sum }}\alpha_{i}^{j}$, where the subscript *G* indicates the genotype. Then, the additive component of the genetic variance is, analogous to the one-locus case above (Equation 7), V_A_ = V(α_*ijkl*_) for the two-locus case and V_A_ = V(α_*G*_) in the general case.

In any case, the regression model in Equation (8) ensures by construction that the additive variance computed from Equation (9) as explained just above is accurate regardless both any dominance and/or epistatic interactions involved in the genetic system and any departures from equilibrium—from HWE and/or from LE—affecting the population frequencies. Indeed, such additive variance accounts for any possible departures from equilibrium frequencies because it is obtained using expressions that involve the genotypic frequencies *p*_*ijkl*_, *i* ≤ *j, k* ≤ *l* (rather than only the marginal ones $p_{ij}^{A},p_{kl}^{B}$, or even the allele frequencies $p_{i}^{A},p_{k}^{B}$, which are the ones used in previous methods).

### Dominance Component

Analogous to the one-locus case (Equation 1), the mean and the additive components of the two-locus regression model (Equation 8) have been detached above in two steps. However, as opposed to the error terms in the one-locus case, which accounted for only dominance interactions, the error terms of the regression model in Equation (8), η_α_, entail all possible interactions together—including also epistasis. Thus, further regression steps are still required for detaching the remaining terms needed for completing the genetic decomposition, leading to a full orthogonal partition of both the genotypic values and the genetic variance. Specifically, the next step consists in detaching the dominance component, δ, from the error terms of Equation (8), η_α_. This can be done with regression η_α_ = **N**_δ_δ+η_δ_, expanding to

(11) $$\left(\begin{array}{c} \eta_{1111}^{\alpha }\\ \eta_{1211}^{\alpha }\\ \eta_{2211}^{\alpha }\\ \eta_{1112}^{\alpha }\\ \eta_{1212}^{\alpha }\\ \eta_{2212}^{\alpha }\\ \eta_{1122}^{\alpha }\\ \eta_{1222}^{\alpha }\\ \eta_{2222}^{\alpha } \end{array}\right)=\left(\begin{array}{llllll} 1 & 0 & 0 & 1 & 0 & 0\\ 0 & 1 & 0 & 1 & 0 & 0\\ 0 & 0 & 1 & 1 & 0 & 0\\ 1 & 0 & 0 & 0 & 1 & 0\\ 0 & 1 & 0 & 0 & 1 & 0\\ 0 & 0 & 1 & 0 & 1 & 0\\ 1 & 0 & 0 & 0 & 0 & 1\\ 0 & 1 & 0 & 0 & 0 & 1\\ 0 & 0 & 1 & 0 & 0 & 1 \end{array}\right)\left(\begin{array}{l} \delta_{11}^{A}\\ \delta_{12}^{A}\\ \delta_{22}^{A}\\ \delta_{11}^{B}\\ \delta_{12}^{B}\\ \delta_{22}^{B} \end{array}\right)+\left(\begin{array}{c} \eta_{1111}^{\delta }\\ \eta_{1211}^{\delta }\\ \eta_{2211}^{\delta }\\ \eta_{1112}^{\delta }\\ \eta_{1212}^{\delta }\\ \eta_{2212}^{\delta }\\ \eta_{1122}^{\delta }\\ \eta_{1222}^{\delta }\\ \eta_{2222}^{\delta } \end{array}\right)$$

Design matrix **N**_δ_ just indicates the dominance deviations associated to each genotype, at each locus. The rows of **N**_δ_ can be obtained with all possible repetitions of the rows of the identity matrix of the appropriate dimension, 3 × 3, in the same way as the two-locus design matrix **N**_α_ in the regression model in Equation (8) are repetitions of rows of the single locus design matrix **N** in the regression model in Equation (1). This analogy makes special sense by rewriting the one-locus regression model (Equation 1) as *G* = **1**μ+**N**α+**I**δ. In the general case, with multiple multiallelic loci, the number of rows combined would equal the number of loci and the dimension of each identity matrix would equal the number of marginal genotypes of the corresponding locus.

In any case, the WLS solution to Equation (11) may be obtained with the method used for Equation (9) above, leading to

(12) $$\delta ={\tilde{\text{H}}}_{\delta }\eta_{\alpha },\text{ with }{\tilde{\text{H}}}_{\delta }={\left({\text{U}}_{\delta }{\left({\text{D}}_{\delta }\right)}^{-1}{\text{U}}_{\delta }{\;}^{\prime }\right)}^{-1}{\text{N}}_{\delta }{\;}^{\prime }\text{P},$$

where **D**_δ_ and **U**_δ_ are built with the non-nil eigenvalues of matrix **N**_δ_′**PN**_δ_ and with their corresponding eigenvectors, respectively. The error term is obtained once again as in Equations (4, 10) as

(13) $$\begin{array}{r} \eta_{\delta }={\text{M}}_{\delta }\eta_{\alpha }, \end{array}$$

with **H**_δ_ = **N**_δ_${\tilde{\text{H}}}_{\delta }$ and **M**_δ_ = **I**–**H**_δ_, **I** being the identity matrix with the appropriate dimension, 9 × 9.

As pointed out above for the multilocus additive components of the genotypic values, having a look at Equation (11) makes it evident that the dominance components of the genotypic values are δ_*ijkl*_ = $\delta_{ij}^{A}+\delta_{kl}^{B}$, *i* ≤ *j, k* ≤ *l*. In the general case (with *n*_*k*_ alleles at the *k*^th^ locus, *k* = 1,…,*l*) they would be δ_*G*_ = $\overset{l}{\underset{k=1}{\sum }}\overset{n_{k}}{\underset{i\leq j=1}{\sum }}\delta_{ij}^{k}$. The dominance variance is thus V_D_ = V(δ_*ijkl*_) or, in general, V_D_ = V(δ_*G*_). This dominance variance is by construction orthogonal to the additive variance provided above through Equation (9), since it comes from the error terms of Equation (8). Furthermore, this dominance component is also accurate regardless both dominance, epistasis and departures from HWE and from LE that may occur, in the same way as justified in relation to the additive variance above.

### Epistasis Components

After having detached both the additive and the dominance components, the remaining error terms in Equation (11), η_δ_, account only for the between-locus interactions (i.e., pairwise epistasis) or for also higher order interactions in the general case. Hence, the epistasis variance is just the variance of those error terms. We may thus call ε = η_δ_ and express the epistatic variance as V_I_ = V(ε_*ijkl*_) for two loci or V_I_ = V(ε_*G*_) in the general case. Once again, orthogonality holds by construction since the epistasis terms so obtained are necessarily orthogonal both to the dominance component, from which they have been detached as error terms in Equation (11), and to the additive component, from which they had previously been detached within the error terms of Equation (8). As a consequence of orthogonality, the sum of the three variance components (additive, dominance, and epistasis) equals the genetic variance and, hence, the epistatic variance can also be expressed as V_I_ = V_G_ − (V_A_+V_D_).

With this, we have achieved the main goal of this paper—to overcome the established misconception that LD precludes orthogonal genetic variance decomposition into additive, dominance and epistasis components. In what follows we nevertheless briefly describe how to further split the epistasis terms, ε, into their natural components (arising as the different groups of interactions of the previous variables) in the face of LD. To do so, additional regressions must keep on being solved sequentially, the first of which detaches the additive-by-additive (AA) component, ε_αα_, as

(14) $$\begin{array}{r} \varepsilon ={\text{N}}_{\alpha \alpha }\varepsilon_{\alpha \alpha }+\eta_{\alpha \alpha },\text{ with }{\text{N}}_{\alpha \alpha }={\text{N}}_{\alpha }⊗{\text{N}}_{\alpha }, \end{array}$$

where ⊗ stands for the Kronecker product—the operator providing interaction terms in regressions (e.g., Harville, 1997; Draper and Smith, 1998). The WLS solution to this regression may be obtained, analogous to Equations (3, 4), in the conventional way as

(15) $$\varepsilon_{\alpha \alpha }={\tilde{\text{H}}}_{\alpha \alpha }\varepsilon ,\text{ with }{\tilde{\text{H}}}_{\alpha \alpha }={\left({\text{N}}_{\alpha \alpha }{\;}^{\prime }\text{P}{\text{N}}_{\alpha \alpha }\right)}^{-1}{\text{N}}_{\alpha \alpha }{\;}^{\prime }\text{P}.$$

The error terms may in their turn be expressed as

(16) $$\begin{array}{r} \eta_{\alpha \alpha }={\text{M}}_{\alpha \alpha }\varepsilon , \end{array}$$

with **H**_αα_ = **N**_αα_${\tilde{\text{H}}}_{\alpha \alpha }$ and **M**_αα_ = **I**–**H**_αα_, using the identity matrix **I** with the appropriate dimension. The next step detaches the dominance-by-additive (DA) and the additive-by-dominance (AD) components, ε_δα_ and ε_αδ_, respectively, as

(17) $$\begin{array}{l} \eta_{\alpha \alpha }=\left({\text{N}}_{\delta \alpha }|{\text{N}}_{\alpha \delta }\right){\left(\varepsilon_{\delta \alpha }|\varepsilon_{\alpha \delta }\right)}^{\prime }+\varepsilon_{\delta \delta },\\ \text{ with }{\text{N}}_{\delta \alpha }={\text{N}}_{\alpha }⊗\text{I}\text{ and }{\text{N}}_{\alpha \delta }=\text{I}⊗{\text{N}}_{\alpha }. \end{array}$$

In this expression, the DA and AD components are obtained simultaneously by concatenating their design matrices into one as (**N**_δα_|**N**_αδ_), which for this particular case works the same way as splitting it in two steps—one for obtaining DA and another one for obtaining AD. The WLS solution of the regression in Equation (17) requires again, analogous to Equations (9, 12), to compute the corresponding eigenvectors and eigenvalues (in this case, those of (**N**_δα_|**N**_αδ_)'**P**(**N**_δα_|**N**_αδ_)) to obtain

(18) $$\begin{array}{l} {\left(\varepsilon_{\delta \alpha }|\varepsilon_{\alpha \delta }\right)}^{\prime }={\tilde{\text{H}}}_{\delta \alpha \alpha \delta }\eta_{\alpha \alpha },\text{with}{\tilde{\text{H}}}_{\delta \alpha \alpha \delta }\\ \;={\left({\text{U}}_{\delta \alpha \alpha \delta }{\left({\text{D}}_{\delta \alpha \alpha \delta }\right)}^{-1}{{\text{U}}_{\delta \alpha \alpha \delta }}^{\prime }\right)}^{-1}\\ \;{\left({\text{N}}_{\delta \alpha }|{\text{N}}_{\alpha \delta }\right)}^{\prime }\text{P}. \end{array}$$

The error terms are the dominance-by-dominance (DD) interactions,

(19) $$\begin{array}{r} \varepsilon_{\delta \delta }={\text{M}}_{\delta \alpha \alpha \delta }\eta_{\alpha \alpha }, \end{array}$$

with **H**_δ*ααδ*_ = $\left({\text{N}}_{\delta \alpha }\;|\;{\text{N}}_{\alpha \delta }\right){\tilde{\text{H}}}_{\delta \alpha \alpha \delta }$ and **M**_δ*ααδ*_ = **I**–**H**_δ*ααδ*_.

It is worth noting at this point that the regression in Expression 17 may as well be performed in two sequential steps—one for obtaining ε_δα_ using **N**_δα_ and a second one for obtaining ε_αδ_ using **N**_αδ_. Each of these two steps can be solved using the common WLS method (Equation 3) and the results so obtained are the same as in Equations (18, 19). Thus, the regression method we are using in this paper when the regressions lead to non-singular matrices—e.g., when using design-matrix (**N**_δα_|**N**_αδ_)—works in the same way as alternative formulations of the regression that do not lead to non-singular matrices—using **N**_δα_ and **N**_αδ_ sequentially. In any case, the pairwise epistasis components AA, DA, AD and DD of the genotypic values and of the genetic variance may be obtained from Equations (14–19) in a way analogous to the additive and the dominance components obtained from Equations (8–10, 11–13), respectively.

In the completely general case (with multiple multiallelic loci), the occurrence of higher order interactions shall accordingly increase the number of regression steps required for completing the decomposition of the epistasis term. With *l* loci, all *l*(*l*−1)/2 pairwise AA components must be detached first using the design matrices of to the two-locus case and concatenating them as done for the DA and AD components above (Equation 17). All DA and AD components must be detached afterwards in a similar way, followed by all DD components, for which a design matrix built as a concatenation of *l*(*l*−1)/2 identity matrices must be used. Next, all third order components AAA, DAA, ADA, AAD, DDA, DAD, ADD, and DDD must be detached sequentially in a way analogous to the pairwise components—with design matrices built as concatenations of Kronecker products of three marginal effects design matrices. The same process must then be repeated up to the *l*th order, at which the D^*l*^ interactions are the last error terms (as the DD interactions were in the two-locus case).

### General Multilocus Regression Models

The theory developed above for the decomposition of the genotypic values and of the genetic variance under arbitrary departures from both additivity, HWE and LE gets summarized by

(20) $$\begin{array}{r} \text{G}=1\mu +{\text{N}}_{\alpha }\alpha +{\text{N}}_{\delta }\delta +\varepsilon . \end{array}$$

This regression model may be derived in particular from Equations (8, 11) and is meant to be solved in three sequential steps, the first of which is trivial—it consists in just computing the mean-corrected vector of genotypic values. Thus, the solution of the regression model in Equation (20) is very similar to that of the classical one-locus case (Equation 1), which requires one fewer regression step, as well as shorter design matrices.

If the decomposition of the epistasis term is also required, the regression model in Equation (20) must be extended. For a genetic system with two biallelic loci, by just considering also Equations (14, 17) it is easy to derive that extension as

(21) $$\begin{array}{r} G=1\mu +{\text{N}}_{\alpha }\alpha +{\text{N}}_{\delta }\delta +{\text{N}}_{\alpha \alpha }\varepsilon_{\alpha \alpha }+\left({\text{N}}_{\delta \alpha }|{\text{N}}_{\alpha \delta }\right){\left(\varepsilon_{\delta \alpha }|\varepsilon_{\alpha \delta }\right)}^{\prime }\\ \;+\;\varepsilon_{\delta \delta }, \end{array}$$

to be solved using two additional regression steps (five in total, the first of which remains trivial). In the general case, the regression model in Equation (21) must be further extended to accommodate all levels of higher order interactions as explained above.

## Applied Cases

Hereafter we consider two cases of particular evolutionary interest that may be addressed using a genetic system with two biallelic loci, *A* and *B*, with pairwise epistasis—BDM incompatibilities and sign epistasis.

### BDM Incompatibilities

First, we focus on the BDM case (e.g., Dobzhansky, 1937). We consider in particular a population in which alleles *A*_1_ and *B*_1_ are fixed, which splits into two isolated populations that are in their turn invaded by initially neutral mutations *A*_2_ and *B*_2_, respectively. However, as soon as the two populations enter into secondary contact the simultaneous occurrence of alleles *A*_2_ and *B*_2_ in individuals causes a fitness decline. The left-hand side of Table 1 shows the genotype-to-phenotype (GP) map of the BDM case we consider here. At the bottom of the table it is shown that when expressing the BDM GP map in terms of individual-referenced genetic effects from the reference of the genotypic value of *A*_1_*A*_1_*B*_1_*B*_1_–*R* = *G*_1111_—all marginal effects are nil. Nevertheless, it is well-known that the presence of all kinds of epistatsis components (AA, DA, AD, and DD) implies that non-nil marginal effects shall arise both when representing the GP map from different individual-reference points and when analyzing it at the population level. Hence, it is expected that additive, dominance and epistasis variance components are non-nil under many conditions.

**Table 1:**
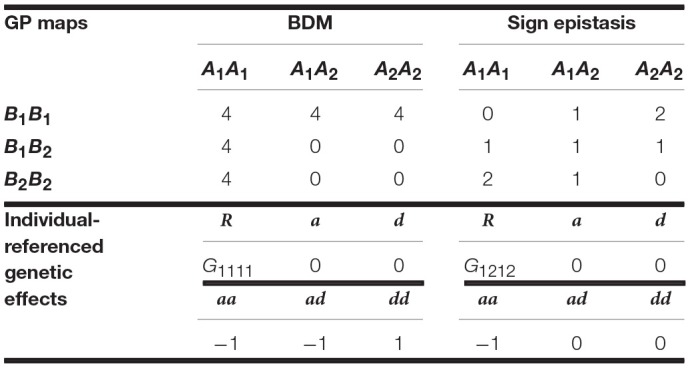
Genotypic values (i.e., GP map) of the BDM and of the sign epistasis cases considered in the text and individual-referenced genetic effects from which they can be built.

*Both cases are locus-symmetric, meaning that the marginal (additive and dominance) individual-referenced effects of loci A and B are equal (i.e., a_1_ = a_2_ = a and d_1_ = d_2_ = d), as well as the individual-referenced pairwise interaction terms da and ad. In both cases, the reference point, R, is chosen in a way that enables to express the GP map with nil marginal effects. For details about the models used to translate between GP maps and individual-referenced (also called functional) genetic effects see (Alvarez-Castro and Carlborg, 2007)*.

**Figure 1:**
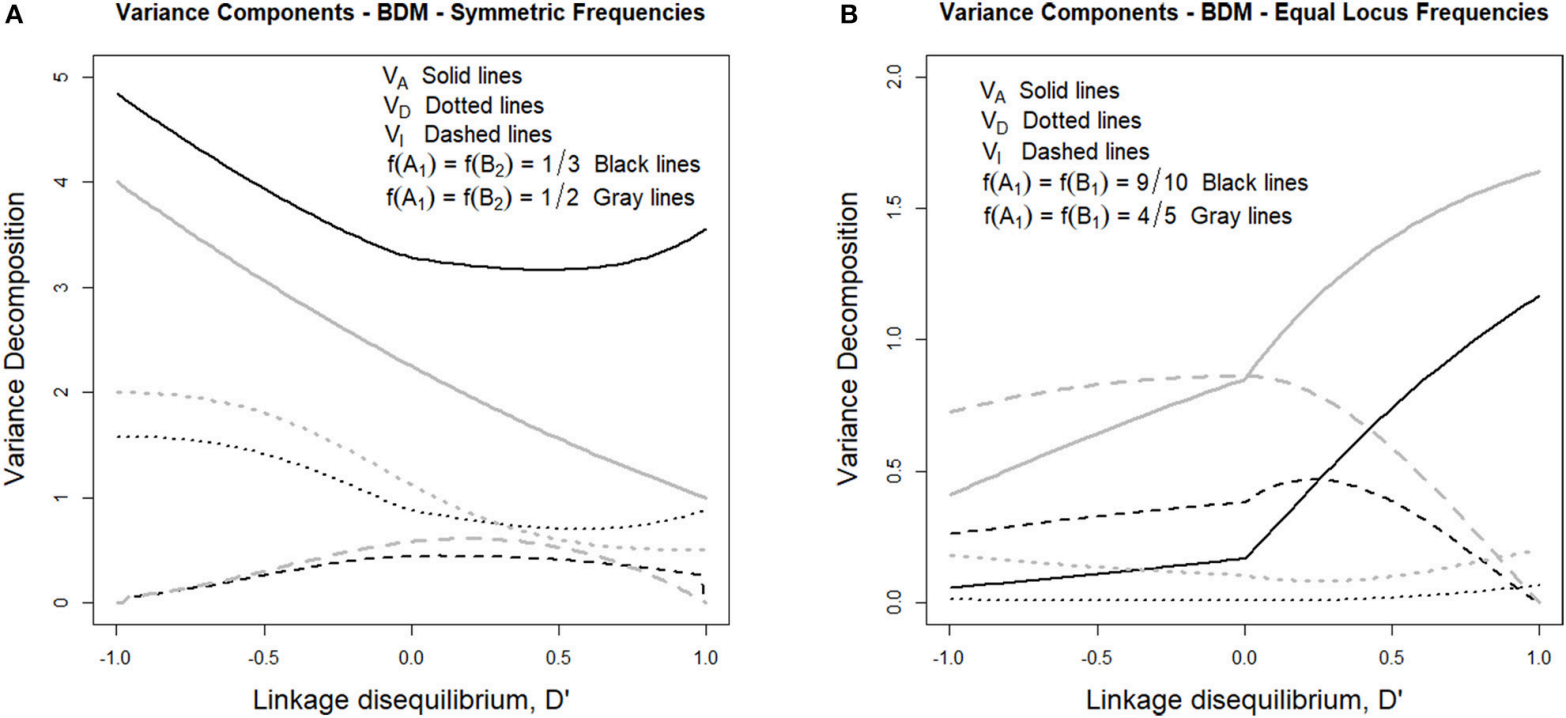
Genetic variance components of the BDM case considered in the text and Table 1, for different sets of allele frequencies and for the full range of possible incidences of LD, measured in terms of the standardized disequilibrium index, *D'*. Additive, dominance and epistasis variances are plotted using solid, dotted and dashed lines, respectively. **(A)** considers two cases with symmetric allele frequencies across loci, *f* (*A*_1_) = *f* (*B*_2_) = 1/3 (black lines) and *f* (*A*_1_) = *f* (*B*_2_) = 1/2 (gray lines). **(B)** considers two cases with equal allele frequencies across loci, *f* (*A*_1_) = *f* (*B*_2_) = 9/10 (black lines) and *f* (*A*_1_) = *f* (*B*_2_) = 4/5 (gray lines).

Such variance components can be observed in Figure 1A, where the genetic variance decomposition is shown for two sets of allele frequencies under their whole range of possible incidence of LD. Figure 1A considers in particular two cases fulfilling *f* (*A*_1_) = *f* (*B*_2_), which is expected to occur at the beginning of the secondary contact described above. In one case (gray lines) the individuals are assumed to come in equal numbers from the two populations and therefore *f* (*A*_1_) = *f* (*B*_2_) = 1/2, whereas in the other case (black lines) the number of individuals coming from one of the populations doubles that of the other one and therefore *f* (*A*_1_) = *f* (*B*_2_) = 1/3. Alvarez-Castro and Le Rouzic (2015) have observed that under LE, despite the evolutionary importance of epistasis in BDM incompatibilities (potentially leading to speciation), the epistasis variance at secondary contact does not exceed half of the additive variance. Here we extend that result to LD since, as we can see in Figure 1A, the epistasis variances (dashed lines) remain at values below half of their corresponding additive variances (solid lines) not only when *D'* = 0 (i.e., under LE), but also to the right of that point (i.e., under positive LD) and to the left of it (i.e., under negative LD).

The latest case, with negative LD, is the realistic one after secondary contact, when any individual is expected to produce one only type of gametes, either *A*_1_*B*_2_ or *A*_2_*B*_1_, depending upon its population of origin. Starting with LE (with *D'* = 0, at the center of Figure 1A) to increasing negative LD (i.e., toward the left-hand side of the figure) the additive and the dominance variances (solid and dotted lines, respectively) increase. The epistasis variance, on the other hand, decreases. Indeed, LD makes some multilocus genotypic classes to be underrepresented or even absent in the extreme case, which causes the decrease of the epistatic variance but does not work in the same way for the dominance variance because homozygotes as well as heterozygotes of some kind remain even under maximum negative LD—e.g., *A*_1_*B*_2_|*A*_1_*B*_2_, *A*_1_*B*_2_|*A*_2_*B*_1_, and *A*_2_*B*_1_|*A*_2_*B*_1_.

We have also inspected the scenario close to fixation of genotype *A*_1_*A*_1_*B*_1_*B*_1_, which is represented in Figure 1B. That figure shows one case with allele frequencies *f* (*A*_1_) = *f* (*B*_1_) = 4/5 (gray lines) and another one with *f* (*A*_1_) = *f* (*B*_1_) = 9/10 (black lines). As opposed to Figure 1A, in Figure 1B the additive variances decrease with increasing incidence of negative LD. This reveals the extent to which negative LD (which is expected to remain for a number of generations after secondary contact, particularly if the individuals of the two populations do not freely intermingle) is hindering fixation, thus bestowing extra time for speciation to be triggered—e.g., for additional reproductive isolation (as mating preference mechanisms) to evolve.

**Figure 2:**
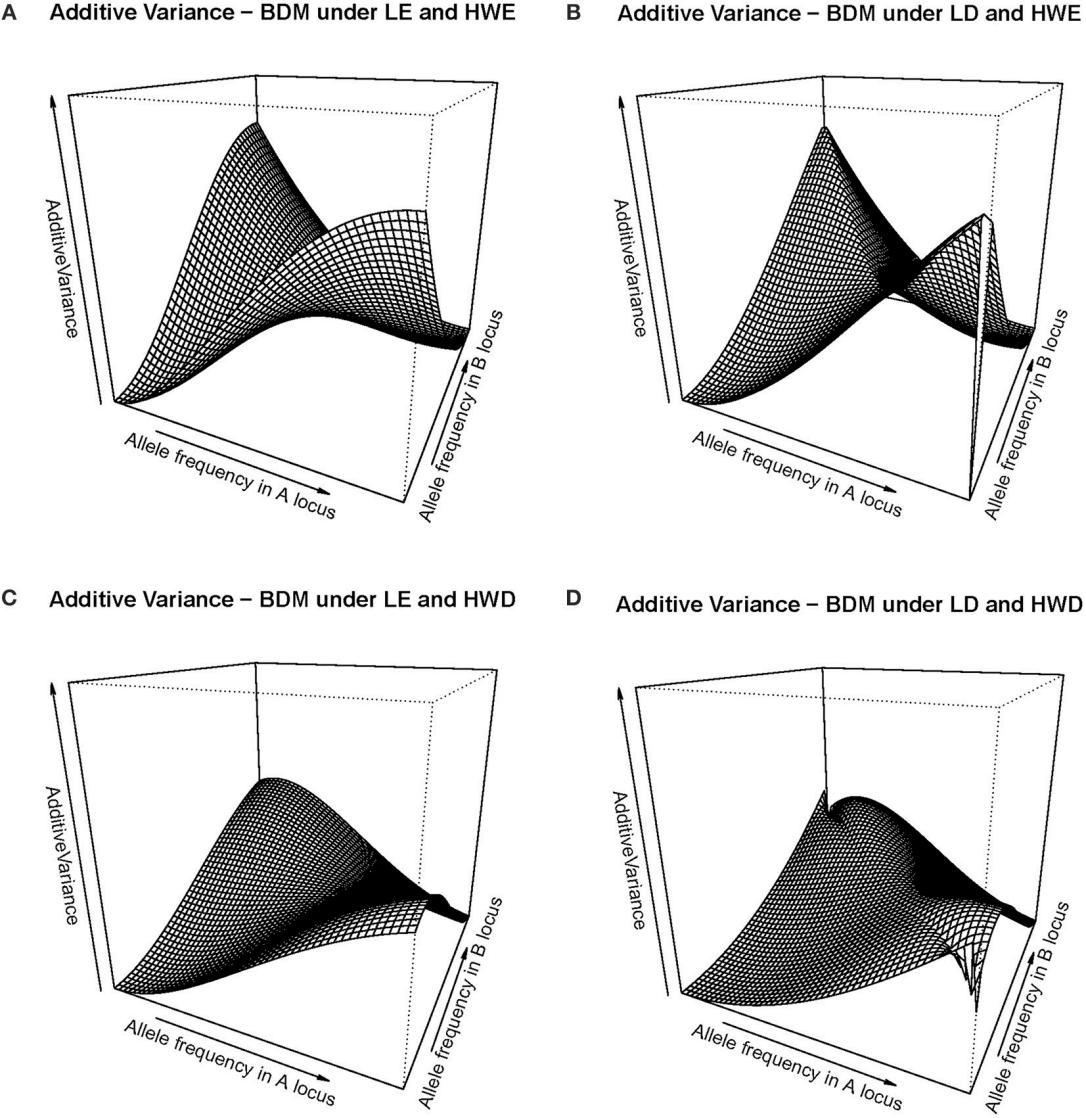
Additive variance surface of the BDM case considered in the text and Table 1, for the whole range of allele frequencies and different incidences of departures from equilibrium frequencies. **(A)** considers the case of equilibrium frequencies. **(B)** considers the case of negative LD with *D'* = −0.6. **(C)** considers the case of a reduction of heterozygotes with a fixation index of *F* = 0.3 at each locus. **(D)** combines both departures **(B, C)** at the same time. In all cases the vertical axis ranges from zero to ten.

That slow down of the selection speed toward fixation due to departures from equilibrium frequencies can also be visualized in Figure 2, which shows several additive variance surfaces of the BDM case here considered. In Figure 2A equilibrium frequencies are assumed. Figure 2B shows a case of negative LD, with a standardized disequilibrium index of *D'* = −0.6. A decrease in additive variance around the fixation of *A*_1_*A*_1_*B*_1_*B*_1_ can be perceived as an incipient plateau toward the left corner of the additive variance surface in Figure 2B, as compared with Figure 1A. That plateau would become much more evident with increasing incidences of negative LD, as Figure 1B demonstrates.

In Figure 2C a different kind of departures from equilibrium frequencies is shown—HWD. Indeed, also a reduction of heterozygotes is expected in the BDM case at secondary contact and as well in the following generations as long as the two populations do not freely intermingle. We have assumed in particular a fixation index of *F* = 0.3 at each of the two loci. That incidence is enough to cause an evident additive variance plateau around the fixation of *A*_1_*A*_1_*B*_1_*B*_1_ (in particular, more evident than in the case of *D'* = −0.6 in Figure 2B). In Figure 2D, with the combined effect of LD (as in Figure 2B) and HWD (as in Figure 2C), the additive variance plateau becomes even larger. Therefore, Figure 2 shows how non-equilibrium frequencies may hamper fixation to occur in a case of BDM incompatibilities, in terms of the changes those departures from equilibrium frequencies (both HWD and LD) cause in the additive variance.

### Sign Epistasis

**Figure 3:**
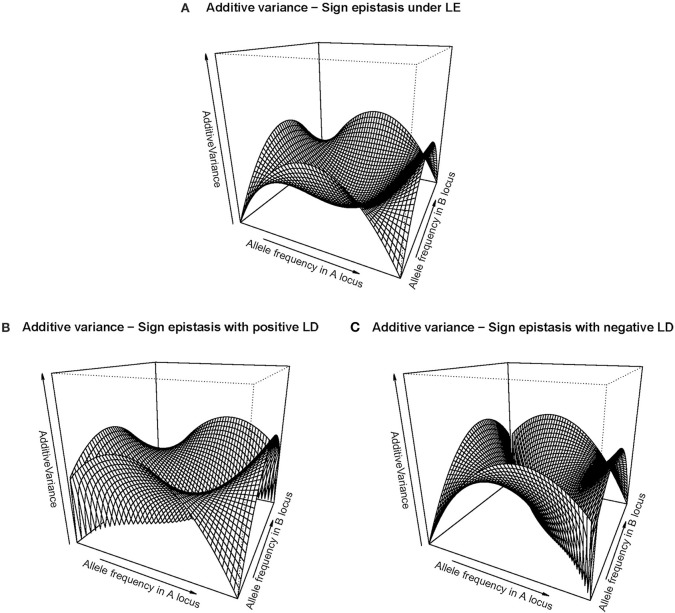
Additive variance surface of the sign epistasis case considered in the text and Table 1, for the whole range of allele frequencies and different incidences of departures from equilibrium frequencies. **(A)** considers the case of equilibrium frequencies. **(B)** considers the case of positive LD with *D'* = 0.9. **(C)** considers the case of negative LD with *D'* = −0.9.In all cases the vertical axis ranges from zero to one.

The right-hand side of Table 1 shows the GP map of the sign epistasis case we consider hereafter. At the bottom of the table it is shown that this case can be built with only AA effects from the reference of *R* = *G*_1212_. Figure 3A shows the well-known surface of additive variance of the sign epistasis GP map under LE (see e.g., Cheverud, 2000; Goodnight, 2015). We recall that result here to compare it with its LD counterpart, which we can plot using the theory provided above. In particular, Figure 3B shows the additive variance surface of sign epistasis with strong positive LD (*D'* = 0.9).

The drop of additive variance at intermediate frequencies in Figure 3A adopts the shape of a sharp ridge in Figure 3B. In the well-studied LE case, the additive variance falls down to zero at intermediate frequencies because of an unstable internal equilibrium, which causes an evolutionary plateau around it (Goodnight, 2015). Evolutionary plateaus are temporary significant decays of selection response between two periods of phenotype change and they are a natural outcome of epistasis (Alvarez-Castro and Le Rouzic, 2015; Goodnight, 2015; Le Rouzic and Álvarez-Castro, 2016). In the BDM case above, we have also commented on an additive variance plateau, although we consciously avoided calling it evolutionary plateau because the decay of selection response would in that case not eventually be followed by a new period of patent selection response—it would instead lead either to a slow pace toward fixation or to speciation. In any case, the ridge of Figure 3B clearly indicates that LD modifies the effect of the evolutionary plateau in the sign epistasis GP map, since it shows that the region affected by such plateau turns into an elongated zone of multilocus frequencies—with similar frequencies of *A*_1_ and *B*_1_. Negative LD causes the same effect but in the perpendicular direction (Figure 3C).

**Figure 4:**
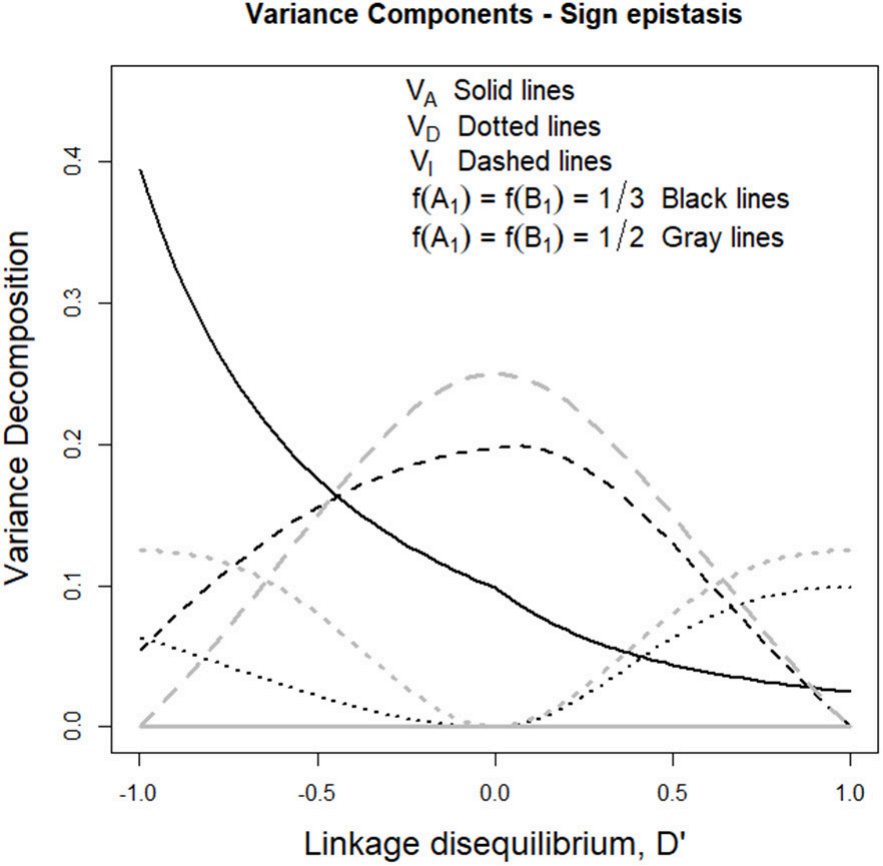
Genetic variance components of the sign epistasis case considered in the text and Table 1, for two sets of allele frequencies and for the full range of possible incidences of LD, measured in terms of the standardized disequilibrium index, *D'*. Additive, dominance and epistasis variances are plotted using solid, dotted and dashed lines, respectively. The two cases considered have equal allele frequencies across loci, *f* (*A*_1_) = *f* (*B*_2_) = 1/3 (black lines) and *f* (*A*_1_) = *f* (*B*_2_) = 1/2 (gray lines).

The aforementioned drop of additive variance of the sign epistasis case at intermediate frequencies is also shown in Figure 4. Indeed, with *f* (*A*_1_) = *f* (*B*_1_) = 1/3 the additive variance (black solid line) increases with negative LD and decreases with positive LD, which reflects the formation of a ridge in the additive variance surface—growing either in the direction of equal allele frequencies in the two loci (with positive LD) or in the perpendicular direction (with negative LD) as mentioned above. Figure 4 also shows that under equal allele frequencies (gray lines), the additive variance remains nil along the whole range of *D'* values.

Beyond what LD implies when sign epistasis occurs, this case also enables us to describe a general property of epistasis with LD, which entails a remarkable difference with systems under LE. Such property can be revealed by focusing on the dominance variances of Figure 4 (dotted lines), which are non-nil despite the absence of dominance in the GP map (recall Table 1). AA effects are known to affect marginal additive effects under LE—indeed, marginal effects of the sign epistasis case we consider here are nil when expressed from the reference of *A*_1_*A*_2_*B*_1_*B*_2_ and also in an F_2_ population but not when frequencies are not intermediate at least at one locus. However, AA effects cannot typically (under LE) generate (or, in general, affect) marginal dominance effects. This is why the dominance variance is nil in Figure 4 when *D'* = 0. However, the combined effect of AA effects and LD makes dominance variance to arise in both cases considered in Figure 4 (either with nil or non-nil additive variance). In what follows, we dissect the mechanism underlying that combined effect.

Let us begin by considering the most extreme cases of LD. With complete positive association of alleles, only three out of the nine genotypes remain—*A*_1_*B*_1_|*A*_1_*B*_1_, *A*_1_*B*_1_|*A*_2_*B*_2_, and *A*_2_*B*_2_|*A*_2_*B*_2_. For the specific pattern of sign epistasis we are considering here, the genotypic values of these genotypes are those of a single locus with overdominance—in particular, 0, 1, and 0, respectively, as Table 1 shows. With complete negative association of alleles, the genotypic values would display underdominance instead (only genotypes *A*_1_*B*_2_|*A*_1_*B*_2_, *A*_1_*B*_2_|*A*_2_*B*_1_, and *A*_2_*B*_1_|*A*_2_*B*_1_ would remain, with genotypic values 2, 1, and 2, respectively). Thus, regardless its sign, LD gradually transforms a two-locus genetic system with sign epistasis (with only AA epistasis and no dominance) into a single-locus system with dominance. That is exactly what the decomposition of the genetic variance with LD (performed as developed above) reflects in Figure 4, with nil dominance variances at *D'* = 0 and with increasing dominance variances (and decreasing epistasis variances) toward both sides. Overall, as opposed to what occurs under LE, LD makes AA epistasis to influence marginal dominance effects. Besides, by generating dominance and dominance variance, the combination of AA effects plus LD also generates all three remaining kinds of epistatic interactions—DA, AD, and DD. Hence, in particular, the epistasis variances shown in Figure 4 include contributions from those three epistasis components, as well as from the AA component.

## Discussion

Throughout one century, the theory for the orthogonal decomposition of the genetic variance into additive, dominance and epistasis components remained unfinished. Several recent works have considered LD when performing genetic variance decomposition, although not providing an orthogonal decomposition of the genetic variance with LD (see Yang, 2004; Mao et al., 2006; Wang, 2011 and references therein; Hill and Mäki-Tanila, 2015 and references therein). Here, we have actually provided an orthogonal decomposition of the genetic variance with LD and thus completed that theory at a time when that has been claimed not to be possible. Our implementations are developed in a way that generalizes previous developments so that the resulting theory accounts for arbitrary numbers of loci and alleles with arbitrary within- and between-/among-locus interactions and under arbitrary departures from equilibrium frequencies. In what regards the latest, it is worth noting that the theory developed in this paper succeeds in attaining an orthogonal decomposition of the genetic variance under completely arbitrary genotypic frequencies, this is to say, actually beyond those that arise from implementing LD through the standardized equilibrium index, *D*' (equivalently through the equilibrium index, *D*), from LE genotypic frequencies.

### Regression Procedures

As recalled by Vitezica et al. (2017), implementing HWD in orthogonal variance decomposition obliged marginal genotypic frequencies to be considered in the developments, as opposed to only allele frequencies (see also Cockerham, 1954; Yang, 2004; Alvarez-Castro and Carlborg, 2007; Álvarez-Castro and Yang, 2011). Similarly, implementing LD obliges multilocus genotypic frequencies to be considered, as opposed to only marginal ones. Hence, unless under LE, an orthogonal decomposition with an arbitrary number of loci cannot be addressed by just combining the results of regressions performed at the single-locus level, which is the approach used in previous models (e.g., Zeng et al., 2005; Alvarez-Castro and Carlborg, 2007; Álvarez-Castro and Yang, 2011)—it has to be addressed instead by means of regressions performed at the multilocus level.

For the implementation of orthogonal genetic variance decomposition with LD to be a coherent extension of the general concept of variance decomposition originally established by Fisher (1918), those multilocus regressions must adhere to the same conceptualization of the regression variables as the classical regression models (e.g., Kempthorne, 1957). Our developments do not only provide the previously known orthogonal decomposition when performed with genotype frequencies under LE, but are in point of fact fully consistent with the rationale of the classical models of orthogonal genetic variance decomposition, which can be shown trough several crucial points.

First, we have designed marginal regression design matrices for multilocus regressions that, although necessarily larger, keep on using the same indexes as the previous single-locus design matrices—indeed, our design matrices for marginal effects are built with rows that are combinations of rows of single-locus design matrices. Second, epistasis keeps on being implemented just as a parameter coming from interactions of the marginal effects—i.e., with design-matrices coming from Kronecker products of marginal design-matrices. Third, we stick to the procedure of sequential regression, detaching each component (mean, additive, dominance and all epistasis types, including both interaction combinations and interaction orders) step by step. Finally, we have shown that the method used to overcome non-singular matrices in WLS regression works as desired since it provides the same solutions as alternative ways of setting out the regression that enable a conventional WLS solution (recall the regression for obtaining AD epistasis).

In general, the theory developed in this paper illustrates the potential of matrix algebra applied to regression analysis in the context of the orthogonal decomposition of the genotypic values and the genetic variance. With two biallelic loci, the regression model providing an orthogonal decomposition into additive, dominance and epistasis components takes a rather simple form even when expanded to show all scalars within each matrix. Then, by just describing the way in which the design matrices must be modified (more precisely, enlarged), the same regression model is straightforwardly extended to arbitrary numbers of alleles and loci. Also by virtue of matrix algebra, the WLS solutions of the subsequent steps of the regression model take the form of manageable expressions even when the conventional WLS solutions involve non-singular matrices.

### Why Has This Decomposition Been Considered Unfeasible?

Some concerns about the feasibility of a fully orthogonal decomposition of the genetic variance with LD come from realizing that, by definition, LD generates non-independence at the among-locus level (e.g., Hill and Mäki-Tanila, 2015). Nevertheless, it should just as well be kept in mind that already at the single-locus level, HWD generate non-independence between/among alleles, which has been reported to prevent the additive components of the genotypic values from fitting to the concept of breeding values—thus loosing part of the properties they have under HWE (as is recalled e.g., by Vitezica et al., 2017). Although that may be considered more or less inconvenient, it does not make the within-locus orthogonal decomposition into additive and dominance genetic components under HWD to be either unfeasible or useless (see e.g., Cockerham, 1954; Alvarez-Castro and Carlborg, 2007; Álvarez-Castro and Yang, 2011; Vitezica et al., 2017). Similarly, considering that LD may make us adjust our interpretation of orthogonal genetic decomposition at the among-locus level does not preclude such decomposition from being both possible (indeed, we have achieved it above) and of significant practical use (as further discussed below).

On the other hand, Zeng et al. (2005) analyzed biallelic models with LD and epistasis and concluded that such conditions make it unfeasible for reduced meaningful models to retain the estimates of genetic effects of a full model. However, the same outcome can occur under other circumstances—e.g., with lack of genotype information (Nettelblad et al., 2012). Thus, again, the fact that orthogonal models do not under all possible circumstances enable meaningful reduced models in which the remaining parameters remain unchanged may be both surprising and inconvenient, but it actually does not mean that the models themselves are neither orthogonal nor biologically meaningful nor useful. Indeed, systems under LD bring about a significant increase in complexity—both conceptually and mathematically—and we have shown above that the orthogonal decomposition of the genetic variance provided in this paper is useful to properly reflect it. In particular, we have used our theoretical results to describe how, under LD, even marginal dominance effects are influenced by AA interactions—a novel feature as compared to systems under LE.

In brief, noteworthy particularities of LD to be kept in mind in what regards orthogonal genetic decompositions have been found out and, although cases for which orthogonal decompositions were attained previously also involve certain particularities, we find it plausible that the specific kind of complexity found to be associated to LD may have made it difficult to imagine how an orthogonal decomposition into additive, dominance and epistastic genetic components under LD could be possible to achieve in theory or how it could be applied in practice.

### Applications

In this paper we have considered two cases of evolutionary interest for illustrating some of the potential uses of our theoretical proposal for orthogonal variance decomposition with epistasis and LD. Although both cases remain at the simplest possible level of two biallelic loci, in the BDM case we have dealt with all types of pairwise epistasis, HWD and LD simultaneously to show how our theory can be used to analyze the emergence of an additive variance plateau by means of which HWD and LD may prevent the fixation of the original genotype after secondary contact. We have also used this case to add up to the fact that (also under LD) epistasis may condition the evolutionary outcome of a genetic system while the epistatic variance remains low.

In the sign epistasis case, we have kept things even simpler by sticking to a GP map that can be built with just AA epistatic effects. It has been by using the simplicity of that genetic system that we have been able to show how LD may (in combination with just AA epistasis) generate dominance variance. More in particular, we have also shown how LD turns the evolutionary plateau that has been described for this system under LE into a ridge, whose orientation depends upon the sign of the standardized disequilibrium index *D'*.

The potential applications of the orthogonal decomposition of the genetic variance under LD certainly go beyond the applications we have here addressed. Álvarez-Castro and Yang (2011) described a method of fitness estimation from equilibrium frequencies at a multiallelic locus under selection, using an accurate expression for the orthogonal decomposition of the genetic variance under HWD. With the theory provided here, that method can now be also applied to multilocus systems (whether multiallelic or not), in which selection shall typically generate departures from LE genotypic frequencies.

We have already mentioned above that, in genetic mapping studies, orthogonal variance decomposition is a key ingredient of model selection strategies. Indeed, the need to overcome difficulties arising in genetic mapping studies and the lack of a satisfactory extension of the classical decomposition of the genetic variance justified the development of alternative orthogonal parameterizations (e.g., Crawford et al., 2017) and even of non-parametric methods (e.g., Gianola et al., 2006). More to the point, it has recently been stressed that several kinds of orthogonal decompositions of the genetic variance can be developed (Huang and Mackay, 2016). However, the best advantages of orthogonality to model selection shall come from genetic models in which the parameters retain the desired biological meaning since those are the ones that make real sense to consider and compare. Such genetic models are the ones Fisher (1918) originally established in his now classical decomposition of the genetic variance.

Incidentally, it has been shown in practice that even when orthogonality is not fully achieved (particularly, due to LD), extensions of the classical models enabling orthogonal decompositions under most of the genetic phenomena involved in the data (particularly, accounting for arbitrary marginal genotypic frequencies, and thus for HWD) provide estimates that are substantially more consistent (e.g., in what regards their genetic meaning) than models that enable orthogonal decompositions only under more restricted conditions (accounting for arbitrary allele frequencies alone; Vitezica et al., 2017). The ideal situation is in any case to count on a fully orthogonal extension of the classical models, which is what motivated the work we are providing in this paper.

Overall, orthogonal genetic variance decomposition is nuclear in evolutionary and quantitative genetics, its usefulness goes nowadays far beyond what Fisher (1918) could possibly envisage when he developed it one century ago and new applications of it keep on surprising us now and again. For instance, advances made in theoretical models of genetic effects and classical variance decompositions about a decade ago (Alvarez-Castro and Carlborg, 2007) have recently been used to improve methods of genomic prediction (Vitezica et al., 2017). Indeed, we find it difficult to set limits today to the actual extent to which the theory provided in this paper may aid the study of evolutionary phenomena and quantitative genetics analyses, particularly in the medium term.

## Author Contributions

JÁ-C established the research aims, conceived the regression models and design matrices, performed the analyses, wrote the paper and obtained funding. RC solved the normal equations, revised the manuscript and obtained funding.

### Conflict of Interest Statement

The authors declare that the research was conducted in the absence of any commercial or financial relationships that could be construed as a potential conflict of interest.

## References

[B1] Álvarez-Castro J. M. (2014). Dissecting genetic effects with imprinting. Front. Ecol. Evol. 2:51 10.3389/fevo.2014.00051 PMC4259164 25583081

[B2] Alvarez-Castro J. M. Carlborg Ö. (2007). A unified model for functional and statistical epistasis and its application in quantitative trait loci analysis. Genetics 176, 1151–1167. 10.1534/genetics.106.067348 17409082 PMC1894581

[B3] Alvarez-Castro J. M. Le Rouzic A. (2015). On the partitioning of genetic variance with epistasis, in Epistasis: Methods and Protocols, eds. Moore J. H. Williams S. M. (New York, NY: Springer, Humana Press), 95–114. 10.1007/978-1-4939-2155-3_6 25403529

[B4] Álvarez-Castro J. M. Yang R.-C. (2011). Multiallelic models of genetic effects and variance decomposition in non-equilibrium populations. Genetica 139, 1119–1134. 10.1007/s10709-011-9614-9 22068562 PMC3247674

[B5] Álvarez-Castro J. M. Yang R. C. (eds.). (2015). Models and Estimation of Genetic Effects. Lausanne: Front Media.

[B6] Bürger R. (2000). The Mathematical Theory of Selection, Recombination and Mutation. Chichester: Wiley.

[B7] Cheverud J. M. (2000). Detecting epistasis among quantitative trait loci, in Epistasis and the Evolutionary Process, eds. Wolf J. B. Brodie E. D. Wade M. J. (Oxford: Oxford University Press), 58–81.

[B8] Cheverud J. M. Routman E. J. (1995). Epistasis and its contribution to genetic variance components. Genetics 139, 1455–1461. 7768453 10.1093/genetics/139.3.1455 PMC1206471

[B9] Cockerham C. C. (1954). An extension of the concept of partitioning hereditary variance for analysis of covariances among relatives when epistasis is present. Genetics 39, 859–882. 17247525 10.1093/genetics/39.6.859 PMC1209694

[B10] Crawford L. Zeng P. Mukherjee S. Zhou X. (2017). Detecting epistasis with the marginal epistasis test in genetic mapping studies of quantitative traits. PLoS Genet. 13:e1006869. 10.1371/journal.pgen.1006869 28746338 PMC5550000

[B11] Dobzhansky T. (1937). Genetics and the Origin of Species. New York, NY: Columbia University Press.

[B12] Draper N. R. Smith H. (1998). Applied Regression Analysis. New York, NY: John Wiley & Sons.

[B13] Fisher R. A. (1918). The correlation between relatives on the supposition of Mendelian inheritance. Trans. Roy. Soc. Edinburgh 52, 339–433.

[B14] Fisher R. A. (1930). The Genetical Theory of Natural Selection. Oxford: Clarendon.

[B15] Franklin R. Gosling R. G. (1953). Molecular configuration in sodium thymonucleate. Nature 171, 740–741. 10.1038/171740a0 13054694

[B16] Galton F. (1886). Regression towards mediocrity in hereditary stature. Anthrop Inst. Great Britain Ireland 15, 246–263. 10.2307/2841583

[B17] Gianola D. Fernando R. L. Stella A. (2006). Genomic-assisted prediction of genetic value with semiparametric procedures. Genetics 173, 1761–1776. 10.1534/genetics.105.049510 16648593 PMC1526664

[B18] Goodnight C. (2015). Long-term selection experiments: epistasis and the response to selection. Methods Mol. Biol. 1253, 1–18. 10.1007/978-1-4939-2155-3_1 25403524

[B19] Hansen T. F. Wagner G. P. (2001). Modeling genetic architecture: a multilinear theory of gene interaction. Theor. Popul. Biol. 59, 61–86. 10.1006/tpbi.2000.1508 11243929

[B20] Harville D. A. (1997). Matrix Algebra From a Statistician's Perspective. New York, NY: Springer.

[B21] Hill W. G. Mäki-Tanila A. (2015). Expected influence of linkage disequilibrium on genetic variance caused by dominance and epistasis on quantitative traits. J. Anim. Breed Genet. 132, 176–186. 10.1111/jbg.12140 25823842

[B22] Huang W. Mackay T. F. (2016). The genetic architecture of quantitative traits cannot be inferred from variance component analysis. PLoS Genet. 12:e1006421. 10.1371/journal.pgen.1006421 27812106 PMC5094750

[B23] Kempthorne O. (1954). The correlation between relatives in a random mating population. Proc. R. Soc. Lond. B. Biol. Sci. 143, 102–113. 13224653

[B24] Kempthorne O. (1957). An Introduction to Genetic Statistics. New York, NY: Wiley.

[B25] Le Rouzic A. Álvarez-Castro J. M. (2016). Epistasis-induced evolutionary plateaus in selection responses. Am. Nat. 188, E134–E150. 10.1086/688893 27860514

[B26] Ma J. Xiao F. Xiong M. Andrew A. S. Brenner H. Duell E. J.. (2012). Natural and orthogonal interaction framework for modeling gene-environment interactions with application to lung cancer. Hum. Hered. 73, 185–194. 10.1159/000339906 22889990 PMC3534768

[B27] Mao Y. London N. R. Ma L. Dvorkin D. Da Y. (2006). Detection of SNP epistasis effects of quantitative traits using an extended Kempthorne model. Physiol. Genomics 28, 46–52. 10.1152/physiolgenomics.00096.2006 16940430

[B28] Nettelblad C. Carlborg Ö. Pino-Querido A. Álvarez-Castro J. M. (2012). Coherent estimates of genetic effects with missing information. Open J. Genetics 2:8 10.4236/ojgen.2012.21003

[B29] Provine W. B. (1971). The Origins of Theoretical Population Genetics. Chicago, IL: University of Chicago Press.

[B30] R Core Team (2017). R: A Language and Environment for Statistical Computing. Viena: R Foundation for Statistical Computing.

[B31] Vitezica Z. G. Legarra A. Toro M. A. Varona L. (2017). Orthogonal estimates of variances for additive, dominance, and epistatic effects in populations. Genetics 206, 1297–1307. 10.1534/genetics.116.199406 28522540 PMC5500131

[B32] Wang T. (2011). On coding genotypes for genetic markers with multiple alleles in genetic association study of quantitative traits. BMC Genet. 12:82. 10.1186/1471-2156-12-82 21936918 PMC3224146

[B33] Watson J. D. Crick F. H. (1953). A structure for deoxyribose nucleic acid. Nature 171, 737–738. 10.1038/171737a0 13054692

[B34] Xiao F. Ma J. Cai G. Fang S. Lee J. E. Wei Q.. (2014). Natural and orthogonal model for estimating gene-gene interactions applied to cutaneous melanoma. Hum. Genet. 133, 559–574. 10.1007/s00439-013-1392-2 24241239 PMC4423532

[B35] Yang R. C. (2004). Epistasis of quantitative trait loci under different gene action models. Genetics 167, 1493–1505. 10.1534/genetics.103.020016 15280257 PMC1470938

[B36] Zeng Z. B. Wang T. Zou W. (2005). Modeling quantitative trait Loci and interpretation of models. Genetics 169, 1711–1725. 10.1534/genetics.104.035857 15654105 PMC1449562

